# Computational Study of Estrogen Receptor-Alpha Antagonist with Three-Dimensional Quantitative Structure-Activity Relationship, Support Vector Regression, and Linear Regression Methods

**DOI:** 10.1155/2013/743139

**Published:** 2013-05-14

**Authors:** Ying-Hsin Chang, Jun-Yan Chen, Chiou-Yi Hor, Yu-Chung Chuang, Chang-Biau Yang, Chia-Ning Yang

**Affiliations:** ^1^Division of Laboratory Medicine, Zuoying Branch of Kaohsiung Armed Forces General Hospital 813, Kaohsiung 81342, Taiwan; ^2^Department of Life Science, National University of Kaohsiung, Kaohsiung 81148, Taiwan; ^3^Department of Computer Science and Engineering, National Sun Yat-sen University, Kaohsiung 80424, Taiwan; ^4^Institute of Biotechnology, National University of Kaohsiung, Kaohsiung 81148, Taiwan

## Abstract

Human estrogen receptor (ER) isoforms, ER*α* and ER*β*, have long been an important focus in the field of biology. To better understand the structural features associated with the binding of ER*α* ligands to ER*α* and modulate their function, several QSAR models, including CoMFA, CoMSIA, SVR, and LR methods, have been employed to predict the inhibitory activity of 68 raloxifene derivatives. In the SVR and LR modeling, 11 descriptors were selected through feature ranking and sequential feature addition/deletion to generate equations to predict the inhibitory activity toward ER*α*. Among four descriptors that constantly appear in various generated equations, two agree with CoMFA and CoMSIA steric fields and another two can be correlated to a calculated electrostatic potential of ER*α*.

## 1. Introduction

Estrogens are critical in the physiology of the female reproductive system, the maintenance of bone density, and cardiovascular health [1, 2]. Estrogen receptors are classified into two isoforms, ER*α* and ER*β*, both of which are members of the nuclear receptor superfamily of ligand-modulated transcription factors [3, 4]. When the natural ligand estradiol or other ligands bind to ER*α*, complex signaling networks lead to a conformational change, specifically in the activation function (AF)-2 helix (H12), allowing estradiol to bind to chromatin; this, in turn, activates or inhibits responsive genes [5, 6]. ER*α* and ER*β* are the targets of pharmaceutical agents used to fight cancers of the reproductive organs, for example, prostate, uterine, and breast cancer [6, 7]. These pharmaceutical agents are divided into three distinct categories: (i) receptor agonists such as 17*β*-estradiol, the estrogen receptor's natural ligand; (ii) antiestrogens, such as the compound ICI 164,384 [5, 8]; and (iii) raloxifene (arylbenzothiophene) [5, 9] and tamoxifen [10], both of which act as agonists as well as antagonists. Raloxifene (compound 25 in Table 1) is a selective estrogen receptor modulator (SERM) providing a safer alternative to estrogen because it is an ER antagonist in mammary tissue and the uterus and also mimics the agonist effects of estrogen on bone and in the cardiovascular system [11]. The U.S. Food and Drug Administration (FDA) recently approved raloxifene for the treatment of osteoporosis [12], and it is also being tested as a preventive drug against breast cancer and coronary heart disease [5, 9]. Because drug resistance and serious side effects, such as venous thromboembolism and fatal stroke, have been reported [13], there is a crucial need for new therapeutic agents. Two major strategies to achieve this are indirect ligand-based and direct receptor-based approaches, both of which could provide a deeper understanding of the structure-activity associations, thereby enabling the development of new compounds with increased activity and selectivity profiles for specific therapeutic targets.

Support vector machine (SVM) is a statistic approach developed for classification and regression. When this tool is applied to the regression, it is commonly called support vector regression for clarity. Because of its prominent prediction and generalization capability, it is widely adopted in various fields. Lately it has been applied to QSAR field in evaluating physicochemical parameters such as solubility, lipophilicity, polarity, and steric properties and further predicting affinity [14–18]. 

The linear regression model seeks a linear combination for input variables **x** ^0^ = (*x*_1_, *x*_2_,…, *x*_*D*_)^*T*^ that best fits the target variable *t*. The model can be formulated as *t* = *y* + *ε* = *w*_0_ + *w*_1_ *x*_1_ + *w*_2_ *x*_2_ + ⋯+*w*_*D*_ *x*_*D*_ + *ε*, where variable *y* is the predicted value and *ε* is the prediction error. The weight parameters *w*_1_, *w*_2_,…, *w*_*D*_ are associated with *x*_1_, *x*_2_,…, *x*_*D*_, and the parameter *w*_0_ imposes an offset on the model. This represents the simplest form for regression.

In this work, a number of models capable of predicting the inhibitory activity of 68 raloxifene derivatives [19] were constructed. 3D-QSAR models, adopting the widely used approaches CoMFA [20, 21] and CoMSIA [22], provide spatially specific pharmacophoric features for future synthesis. 2D-QSAR models on the base of physicochemical descriptors selected by SVR and LR methods were also performed to seek an alternative approach in relating structural features to affinity between ER*α* and the raloxifene derivatives. In all, this information provides clear guidelines for the synthesis of additional compounds accelerating combinatory chemistry in the development of new drugs. 

## 2. Materials and Methods

### 2.1. Data Set and Biological Activity

This study considered 68 compounds of raloxifene derivatives in a core of aryl-benzothiophene [19]. Structural information and bioactivity associated with MCF-7 cells are listed in Table 1. In 3D-QSAR modeling, 56 compounds formed a training set and 10 compounds formed a test set to externally examine the models. Compounds 9 and 37, both with estimated IC_50_ = 1000 nM, were removed because they were always outliers in the training or test set, and retaining them made the models unacceptably unstable. It is likely that their exact IC_50_ values lie somewhere between 600 and 1000 nM. The test set compounds and compounds not included in modeling are marked in Table 1. In SVR and LR modeling, all 68 compounds were included to choose descriptors for model construction.

### 2.2. Structure Preparation and Alignment

**Figure 1:**
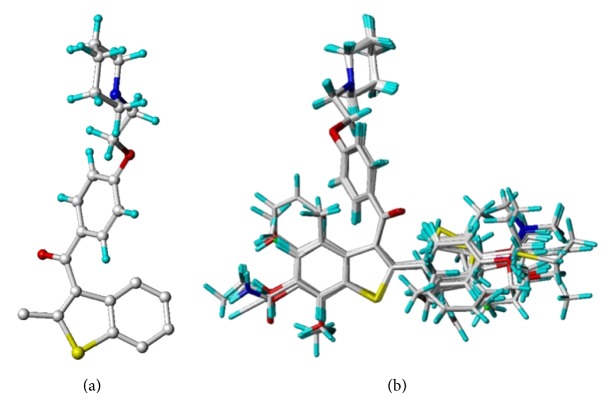
(a) The alignment core used in this study. (b) The result of alignment using align database in Sybyl.

Gasteiger-Hückel charge assignment and a Tripos force field were used to prepare the structure of the compound. The geometry of each arylbenzothiophene derivative was minimized using the simplex algorithm followed by the Powell algorithm to an energy convergence criterion of 0.05 kcal/mol Å. The alignment of compounds is an essential step in determining the structure-activity relationship because the maximized overlap of pharmacophoric features responsible for producing a biological response greatly increases the correlation between structure and activity. A ligand-based approach was adopted in this study, in which each compound in its energetically minimized geometry was aligned according to the core structure, as illustrated in Figure 1(a). The alignment results are given in Figure 1(b). It is notable that the 68 compounds were aligned in 3D space such that most of structural features common to all of the compounds had the same Cartesian coordinates.

### 2.3. CoMFA and CoMSIA

This study used molecular modeling software Sybyl 8.1 (Tripos International, St Louis, MO) for the CoMFA and CoMSIA models. Two CoMFA descriptors, steric (Lennard-Jones 6-12 potential) and electrostatic (Columbic potential) field energies, were calculated using an sp3 carbon atom carrying a +1.0 charge set at default parameters, to serve as a probe atom. In addition to steric and electrostatic fields, CoMSIA also considers hydrophobic and hydrogen bond donor/acceptor interaction. These five similarity indices were calculated using a Gaussian-type distance-dependent function using a default attenuation factor of 0.3. The probe atom was set to the same default parameters used in CoMFA.

Both CoMFA and CoMSIA use pIC_50_ as the target variable in partial least squares (PLS) regression [23] to derive 3D-QSAR models. The predictive value of the model was evaluated by calculating the leave-one-out cross-validated (LOOCV) coefficients, *q* ^2^ [24], using the following equation:

(1) $$\begin{array}{c} q^{2}=1-{\left(\frac{\sum_{Y}{\left(Y_{\text{pred}}-Y_{\text{actual}}\right)}^{2}}{\sum_{Y}Y_{\text{actual}}-\bar{Y}}\right)}^{2}, \end{array}$$

where *Y*_pred_ is predicted affinity (calculated by model), *Y*_actual_ is actual affinity (obtained by experiment), and $\bar{Y}$ is mean actual affinity. The term ∑(*Y*_pred_−*Y*_actual_)^2^ is the predictive sum of squares (PRESS). The number of components giving the lowest PRESS value determines the optimum number of component (ONC) to generate the final PLS regression model. The conventional coefficient, or the non-cross-validated correlation coefficient, *r* ^2^, was subsequently calculated to characterize the statistics of the built model. In general, a *q* ^2^ > 0.5 is an indication of internal predictability [25, 26], whereas an *r* ^2^ > 0.5 indicates that the constructed model is fairly good and interpretative [26, 27]. 

### 2.4. Generation of Physicochemical Molecular Descriptors for 2D QSAR

All chemical structures were generated using Sybyl 8.1 software package, whereas molecular topological indices were generated using Material Studio (Accelrys, San Diego, CA). Overall, Material Studio produced 231 descriptors, including fast descriptors of E state keys, molecular connectivity indices, spatial descriptors, and Jurs descriptors. 

### 2.5. Feature Selection Procedure

The feature selection method for choosing proper descriptors is composed of feature ranking and sequential feature addition or deletion. We adopt the idea of maximal correlation and minimal redundancy. The objective formula is given as follows:

(2) T∗=arg maxT⊆F{1|T|∑fi∈TC(fi,t)−w         ∗2|T|∗(|T|−1)∑fi,fj∈T,fi≠fjC(fi,fj)},

where **T** denotes any feature subset, **T** ^*^ represents the optimal feature subset, *C*(*x*, *y*) denotes the correlation function between variables *x* and *y*, and *F* denotes the universal set consisting of all available features, *F* = {*f*_1_, *f*_2_,…, *f*_*D*_}. The value of *w* is a weight that can be adjusted to represent the relative importance of these two terms.

Since solving **T** ^*^ is an optimization problem, it will inevitably involve a combinatorial search. If an exhaustive search is applied, O(2^|**F**|^) cases should be examined. In order to avoid an exhaustive search, we followed the idea of Peng et al. [28] and adopted a sequential and greedy search approach. We defined the *ranking score* of an unselected feature *f*_*i*_ as

(3) $$\begin{array}{c} S\left(f_{i}\right)=C\left(f_{i},y\right)-\frac{1}{\left|T_{S}\right|}\sum_{f_{j}\in Ts}^{}C\left(f_{j},f_{i}\right), \end{array}$$

where **T**_*s*_ denotes the selected feature subset and *y* denotes the target value. 

After the feature ranking is obtained, the RMSE (root mean square error) $\sqrt{\sum_{i}{\left(y_{i}-t_{i}\right)}^{2}/N}$ was tested by cross-validation in a sequential forward manner. The next step is to locate where the minimal RMSE takes place, say *k*, and select the top *k* ranking features. Subsequently, a sequential feature deletion and a sequential feature addition procedure were applied for *m* rounds. Finally, assuming not too many features are kept, the reserved features are subject to an exhaustive search and export the top *r* feature subsets. The entire procedure is given as follows.

*Procedure: Feature Subset Selection for Regression.*

*Input*. The independent variable is **X** and target variable is **y**. The round number is *m* for sequential feature deletion and addition procedure, and *r* is for the top ranking feature subsets. Assume the linear regression method [29] is adopted to evaluate RMSE. 

*Output*. The top *r* ranking feature subsets from the reserved feature set **T**_*s*_. 

*Step 1*. Apply a sequential search approach to determine the feature ranking.

*Step 2*. Locate the feature subset associated with the minimal RMSE.

*Step 3*. For *i* = 1 to *m* do 

*Step 3.1*. Apply a sequential feature deletion process to the selected features and determine which features are to be removed.

*Step 3.2*. Apply a sequential feature addition process to append unselected features to the selected features and determine which features are to be added.

*Step 4*. Assume the reserved feature set to be **T**_*s*_. Apply an exhaustive search to the reserved features and export the top *r* ranking feature subsets among **T**_*s*_.

## 3. Results and Discussion

### 3.1. Statistics for CoMFA and CoMSIA Models

Listed in Table 2 are the statistic results of 3D-QSAR modeling. We used the partial least squares regression method [23] with the leave-one-out cross-validation procedure [24] to determine the optimum number for the principal components. In the two models created, the leave-one-out cross-validated correlation coefficients (*q* ^2^) all reached the criterion *q* ^2^ ≥ 0.5, and all statistics with the conventional, non-cross-validated correlation coefficients were greater than 0.8. In the CoMFA model, the contributions of steric and electrostatic fields were similar. Because the hydrophobic interaction did not significantly contribute to the CoMSIA model, we removed the hydrophobic descriptor to improve statistical analysis. 

**Figure 2:**
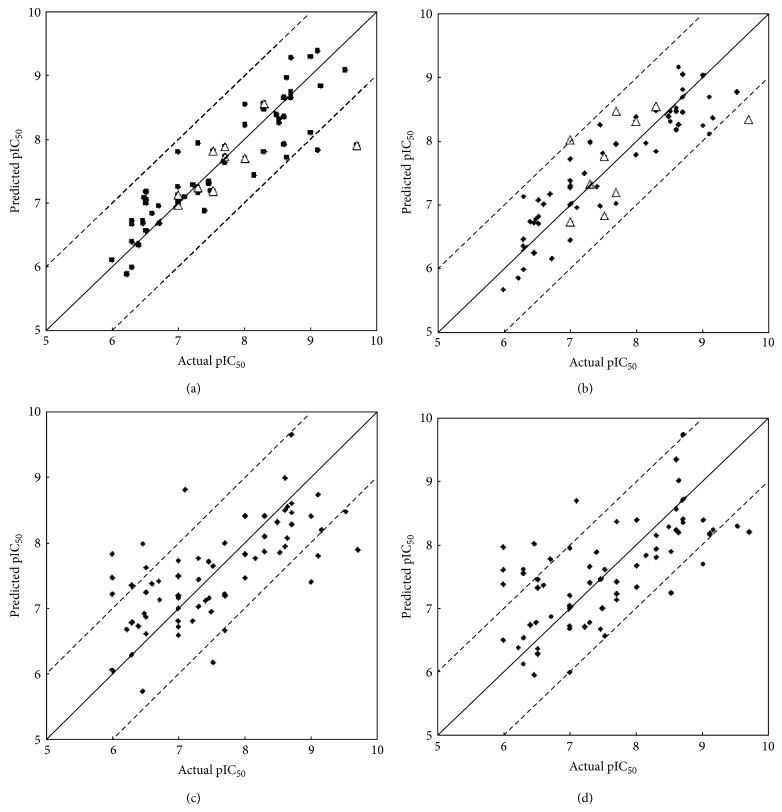
Comparison of actual versus predicted raloxifene inhibitory activity based on (a) CoMFA, (b) CoMSIA, (c) LR, and (d) SVM models. The diagonal in the four plots is the *y* = *x* line, whereas the dashed lines indicate the ±1 log point margins of error for analyses. The solid dots represent the modeling results on training set, whereas the open triangle points in CoMFA and CoMSIA plots represent the test sets.

The predicted pIC_50_ values are listed in CoMFA and CoMSIA columns of Table 1. The predicted and actual pIC_50_ values for training set compounds are plotted in Figures 2(a) and 2(b), for CoMFA and CoMSIA, respectively. To validate our models, we predicted the pIC_50_ for compounds in each corresponding test set (also shown in Figures 2(a) and 2(b)). Most of the absolute residual values, particularly for the training set data points, were less than 1 logarithm unit.

### 3.2. Statistics of SVR and LR Models

The original data set contains 68 instances, each of which consists of one pIC_50_ value and 231 descriptors (features). Since our goal is to use the descriptors to predict the pIC_50_ value, it is reasonable to involve descriptors that are highly correlated with the pIC_50_ value. Any descriptor that has very few distinct values is regarded as invariant to the pIC_50_ value and thus would not facilitate the prediction. The checking method is to calculate the median absolute deviation (MAD), which is given by Med(Abs(**x** − Med(**x**))), where Med and Abs denote median and absolute operators, respectively. There are totally 120 descriptors whose MAD values are equal to zero. Consequently, only 111 descriptors are employed for the subsequent processing. Before performing the regression process, a normalization procedure is applied to the reserved descriptors, that is, $x_{i}^{\prime }=\left(x_{i}^{}-\overset{-}{x}\right)/\sigma_{x}$, where $\overset{-}{x}$ and *σ*_**x**_ represent mean and standard deviation for the descriptor *x*, respectively. 

We applied the feature selection procedure with *m* = 1 on the data set. During the feature selection process, the linear regression was used to evaluate RMSE. Because only 11 descriptors are left for the exhaustive search, we set *r* = 2^11^ − 1 to let the program export all combinations. The intercorrelations between the selected 11 features, as well as the intercorrelations between each feature and pIC_50_, are listed in Table 3.

### 3.3. Steric Fields Determined by CoMFA and CoMSIA Models

**Figure 3:**
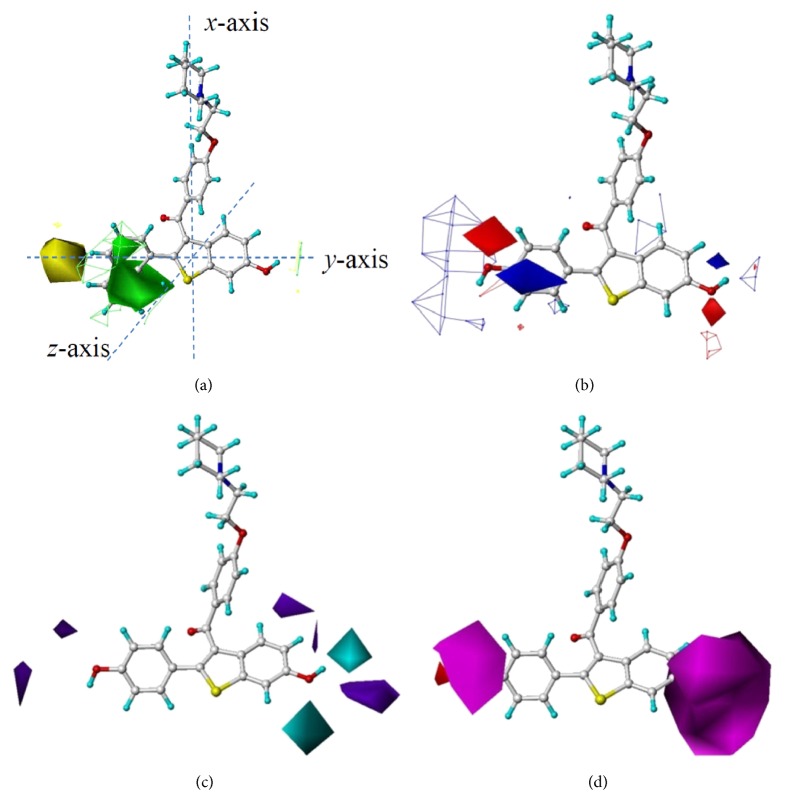
Results obtained by modeling MCF-7 cell inhibitory activity based on 3D QSAR methods. (a) Superimposed steric fields determined by CoMFA (mesh) and CoMSIA (solid) methodologies, in which green contours indicate regions where a relatively bulky substituent would increase inhibitory activity, whereas the yellow contours indicate areas where a bulkier substituent would decrease inhibitory activity. Compound 55 is displayed in the background for reference. The Cartesian coordinate frame is given. (b) Superimposed electrostatic fields determined by CoMFA (mesh) and CoMSIA (solid) methodologies, in which blue contours indicate regions where a positively charged substituent would increase inhibitory activity, whereas the red contours indicate regions where a negatively charged substituent would increase inhibitory activity. Compound 25 is displayed in the background for reference. (c) Hydrogen bond donor field, in which a cyan region favors hydrogen bond donors while a purple region disfavors hydrogen bond donors. Compound 25 is displayed in the background for reference. (d) Hydrogen bond acceptor field, in which a pink region favors hydrogen bond acceptors, while a red region disfavors hydrogen bond acceptors. Compound 25 is displayed in the background for reference.

Figure 3(a) is a superimposed image of two steric fields generated using CoMFA and CoMSIA on MCF-7 cell inhibition. Both steric models indicate that the regions around C2′ and C3′ are steric-favorable. This explains why the activity of compound 55 (IC_50_ = 0.8 nM), the 1′-naphthyl of which is in contact with the green contour, was 100 times higher than that of compound 66 (IC_50_ = 80 nM), the 2′-naphthyl of which is not in contact with the green contour but in contact with a steric-unfavorable region in yellow. Likewise, compound 59 (IC_50_ = 3 nM and an isopropyl group to replace the phenyl ring) was more potent than compound 64 (IC_50_ = 20 nM and a smaller ethyl group to replace the phenyl ring); compound 24 (IC_50_ = 2.5 nM) was more active than compound 34 (IC_50_ = 100 nM, with a phenyl group on C4′ and being in contact with the yellow steric-unfavorable contour). Near C6 a steric-favorable contour was observed in CoMFA. This tiny green contour explains why compound 4 (IC_50_ = 20 nM, with an ethynyl group on C6) was more active than compound 8 (IC_50_ = 300 nM, with a methyl group on C6).

### 3.4. Electrostatic Fields Determined by CoMFA and CoMSIA Models

Figure 3(b) shows two electrostatic fields generated by CoMFA and CoMSIA. Although the two electrostatic models were not identical, there was no conflict. In CoMSIA an electronegativity favorable red contour surrounds the phenyl moiety, indicating that a heteroatom with a partial negative charge would have a positive effect on inhibitory activity. This explains why compounds 27 (IC_50_ = 1 nM, with a chlorine) and 28 (IC_50_ = 2.3 nM, with a fluorine) are more active than compound 33 (IC_50_ = 50 nM, with a methyl group). In the vicinity of the CoMSIA's red contour, a blue contour was observed in CoMFA. Together these two contours suggest that a hydroxyl group attached to C4′ increases activity. 

Both CoMFA and CoMSIA show a contour favorable to a negative charge near C6 and a contour favorable to a positive charge farther away, indicating that a hydroxyl group herein would increase activity. The activities of compounds 25 (IC_50_ = 0.2 nM, with a hydroxyl group), 4 (IC_50_ = 20 nM, with an ethynyl group), and 8 (IC_50_ = 300 nM, with a methyl group), differing in the substituent on C6, varied according to this electrostatic feature.

### 3.5. Hydrogen Bond Donor and Acceptor Fields Determined by CoMSIA Model

Preferences of hydrogen bond donors and acceptors are presented in Figures 3(c) and 3(d), respectively. A number of hydrogen bond donor favorable/unfavorable contours are in the vicinity of C6 (Figure 3(c)). The activity of compound 25 (IC_50_ = 0.2 nM), whose hydroxyl hydrogen atom is in contact with one cyan contour, is higher than that of compound 8 (IC_50_ = 300 nM), with a methyl group on C6.

Two hydrogen bond acceptor favorable contours surround C4′ and C6. Accordingly, compound 28 (IC_50_ = 2.3 nM, with a fluorine on C4′) is more potent than compound 33 (IC_50_ = 50 nM, with a methyl group on C4′), and compound 7 (IC_50_ = 250 nM, with a methoxy group on C6) is slightly more active than compound 8 (IC_50_ = 300 nM, with a methyl group on C6). Meanwhile, the characteristic of favoring hydrogen bond acceptors near C4′ and C6 confirms the red electronegative contours in Figure 3(b).

### 3.6. Projecting CoMSIA Fields onto ER*α* Binding Pocket Determined by X-Ray Crystallography

**Figure 4:**
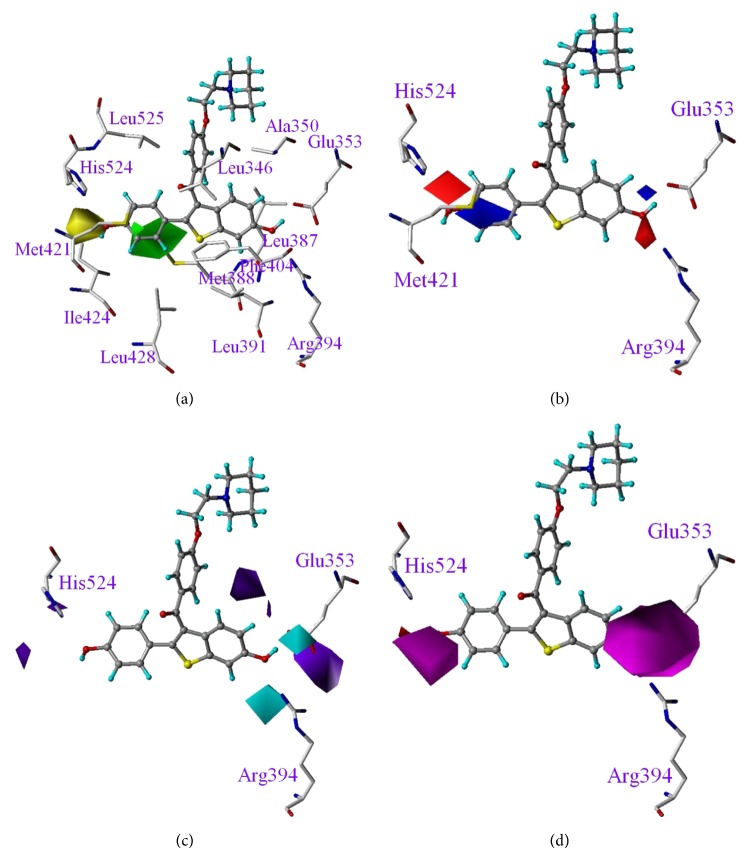
Overlay of CoMSIA fields onto ER*α* binding cavity. (a) Steric, (b) electrostatic, and (c) hydrogen bond donor and (d) hydrogen bond acceptor fields. Color codes are the same as specified in Figure 3. Raloxifene is displayed in the background for reference.

In Figure 4, we superimposed CoMSIA fields onto the activity site of ER*α* (PDB code: 1ERR) [30] to reveal the correlation between the observed fields and ER*α*'s amino acids involved in the binding of modulators. The raloxifene structure used in our 3D-QSAR modeling was obtained by energy minimization and therefore was slightly different from the ER*α* bound that one retrieved from PDB (1ERR). The RMSD between the two raloxifene structures is 0.66 Å, with a minor deviation caused by the orientation of the long chain extended from C3. Since the contour maps in CoMFA and CoMSIA models are about the phenyl and benzothiophene moieties, projecting the contour maps onto the ER*α* binding cavity for discussion is proper. As shown in Figure 4(a), the green, steric favorable contour matches the empty area around Leu525 and Leu428, whereas the yellow, steric unfavorable contour corresponds to the corner surrounded by residues of His524, Ile424, and Met421. In Figure 4(b), the negative and positive charge favorable contours on C6 point toward the positively charged guanidinio of Arg394 and negatively charged carboxylic group of Glu353, respectively. Moreover, the blue contour above the phenyl ring moiety is related to the C4′ red contour. That is, a reduction in phenyl ring electronegativity caused by the electron-withdrawing heteroatom adjacent to C4′ benefits the interaction of the inhibitor and ER*α*. Consequently, the resulting positive charge of the phenyl ring increases the interaction between the inhibitor and Met421 sulfur atom, carrying a partial negative charge. Such electrostatic attractions help discriminate the binding of the inhibitor to ER*α* from ER*β*, as pointed out earlier in Salum's CoMFA model in ligand binding selectivity over ER*α* and ER*β* [31]. ER*α* and ER*β* isoforms share an overall 58% sequence identity in binding domain, particularly their ligand-binding cavities, which differ by only two amino acids of highly conserved characteristics—Leu384 and Met421 on ER*α* and Met336 and Ile373 on ER*β*. Met421 in ER*α* and Ile373 on ER*β* are highly involved in the accommodation of ligands, and are regarded as pivotal in the process of selectivity [32, 33].  Figure 4(c) shows that the contour near C6 favorable to the hydrogen bond donor points toward the carboxylic oxygen atoms of Glu353. In Figure 4(d), the contour favorable to the hydrogen bond acceptor on C6 points toward the guanidinio hydrogen atoms of Arg394 and a contour favorable to the hydrogen bond acceptor on C4′ points toward His524 amide hydrogen. In all, the hydroxyl groups located on C4′ and C6 in conjunction with the residues of Glu353, Arg394, and His524 were demonstrated to form a stable hydrogen bonding network.

### 3.7. SVR and LR Results

For the previously mentioned selected 11 features, totally 2047 cases were to be examined. We applied the linear regression and leave-one-out cross validation (LOOCV) techniques to evaluate all the 2047 cases. The upper part of Table 4 gives the top 10 feature subsets, including formulas and the corresponding LOOCV RMSEs, based on LR. The feature subsets are ranked in terms of RMSEs. It is shown that the best RMSE is 0.7364, which is associated with eight features. To compromise between the model complexity and prediction capability, we adopted the 7th LR model equation, which consists of six features and whose RMSE is 0.7484, to demonstrate the prediction of pIC_50_ listed in LR column, Table 1.  Figure 2(c) plots the actual pIC_50_ values against the predicted values based on this model equation.

In addition to the linear regression (LR), we also applied the linear support vector regression (SVR) [34, 35] to all 2047 feature subsets. The top 15 feature subsets, including formulas and the corresponding LOOCV RMSEs, are listed in the lower part of Table 4. The model equation with the lowest RMSE = 0.7104 is characterized by nine features. To compromise between the model complexity and prediction capability, we adopted the 4th SVR model equation, with five features and RMSE = 0.7273, to demonstrate the prediction of pIC_50_ listed in SVR column, Table 1.  Figure 2(d) plots the actual pIC_50_ values against the predicted values according to this equation. Comparison between the results of LR and SVR suggests that SVR is superior to LR. 

### 3.8. Comparison of SVR Prediction with CoMFA and CoMSIA Models

Models equations derived from LR and SVM approaches indicate that a number of features consistently provide contributions in determining the target variable. Variables *x*_6_, *x*_7_, *x*_9_, and *x*_11_ appear in all the derived equations in both SVM and LR models, whereas variables *x*_3_ and *x*_4_ appear in most of the derived equations. Meanwhile, variables *x*_6_, *x*_9_, and *x*_11_ are quite stable in being positively correlated to the target variable, while *x*_3_, *x*_4_, and *x*_7_ are negatively correlated to the target variable. Variable *x*_6_ represents the molecular shadow area projected on *ZX* plane; variable *x*_7_ represents the shadow area fraction on *YZ* plane. These two descriptors are found in accordance with CoMFA and CoMSIA steric fields shown in Figure 3(a), where the Cartesian coordinate frame is specified. The positive sign of *x*_6_ coefficients in the derived equations suggests that an increase in molecular shadow area on *ZX* plane enhances inhibitory activity, and this is in agreement with Figure 3(a)'s green, steric-favorable contours. That is, along the *y*-axis point-of-view, the shadow area on *YZ* plane can be extended by adding bulky groups in contact with the green contours. Compound 55 (IC_50_ = 0.8 nM) with 1′-naphthyl modification is a good example. Variable *x*_7_, the shadow area fraction on *YZ* plane, is negatively correlated to inhibitory activity and can be correlated to the yellow steric-unfavorable contour in Figure 3(a). That is, an elongated side chain attached on C4′ would increase the shadow area projected on *YZ* plane (which can be seen with a view point along the *x*-axis) and reduce the activity. 

**Figure 5:**
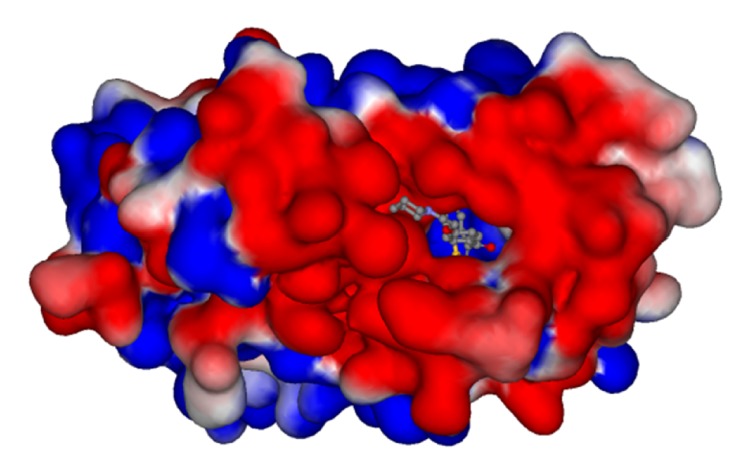
Electrostatic potential on the ER*α* protein surface around the active site of raloxifene (PDB code: 1ERR). Electronegative and electropositive charges are colored in red and blue, respectively.

Variables *x*_9_, the dipole moment about the *z*-axis, and *x*_11_, a Jurs descriptor that is associated with relative positive charge surface area, are both positively correlated to the activity. Analysis on dipole moment about *z*-axis shows that compounds with positive values possess higher activity, which implies that the activity can be boosted by positive charges distributed on compound surface. Together, features *x*_9_ and *x*_11_ suggest that the positive electrostatic potential benefits the inhibitory activity. These findings could be related to the electronegativity of the gate of ER*α* binding pocket (Figure 5) in which an inhibitor with a partial positive charge enters more easily. The electrostatic potential shown in Figure 5 is based on the solved X-ray structure in PDB code 1ERR [30]. 

## 4. Conclusion

Our results have shown that the hydroxyl groups on both C6 and C4′ are irreplaceable, due to the strong hydrogen bonding network linking to Glu353 and Arg394 on C6 side and to His524 on C4′ side. Accordingly, compounds 25 (raloxifene), 26, 45, and 55, possessing two hydroxyl groups at C4′ and C6 sites, have satisfactory IC_50_ values. Earlier results from the literature showed that in cases of E1 (estrone), E2 (17*β*-estradiol), and E3 (estriol) replacing the hydroxyl groups with methoxy eliminated the affinity toward both ER*α* subtypes [36–38]. Likewise, compounds 7, 20, 21, 22, and 23 with a methoxy group on C6 held poor IC_50_ values because of disruption to the hydrogen bond network and steric disfavor. 

Comparison of RMSEs among different feature combinations suggests that if all 231 features are adopted for regression, the RMSEs are not good. On the other hand, if the appropriate feature selection method is used, the performance gets improved. From the results, we can see that most of the RMSEs obtained by SVR outperform those of the LR. This may be attributed to the well-selected features and prominent prediction capability of SVR, because the selected features are not specialized to the evaluation method. In summary, the best RMSE is 0.7580 when ten features are adopted to perform SVR. If the subsets of only 5 features are considered, the best RMSE of SVR is 0.7273.

In the present study models built on different methods were successfully employed to gain detailed insights on the structure of ER*α* modulators. Accordingly, the clues derived from contour analysis can be used for further design work based on arylbenzothiophene and for screening large chemical databases for compounds with potential ER*α* activity.

## Figures and Tables

**Table 1:** Structures, experimental activities presented in IC_50_ and pIC_50_ values, and predicted pIC_50_ values by different modeling approaches.

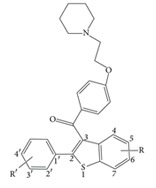			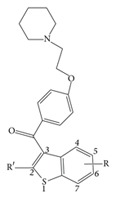					
1–54			55–68					

No.	Substituents		IC_50_ (nM)	pIC_50_	Predicted			
	R	R′			CoMFA	CoMSIA	LR	SVM

1	H	H	300	6.52	7.17	6.72	6.88	6.39
2	H	4′-OH	35	7.46	7.33	6.98	7.71	7.46
3∗	H	4′-OMe	100	7.00	**7.12**	**6.74**	7.00	6.70
4	6-C*≡*CH	4′-OH	20	7.70	7.64	7.03	7.23	7.15
5∗	6-CO_2_Me	4′-OH	30	7.52	**7.18**	**6.83**	6.17	6.58
6	6-COMe	4′-OH	60	7.22	7.28	7.49	6.81	6.72
7	6-OMe	4′-OH	250	6.60	6.83	7.02	7.38	7.38
8	6-Me	4′-OH	300	6.52	7.05	6.81	7.25	7.33
9∗∗	6-Cl	4′-OH	1000	6.00			7.22	7.39
10	6-CONH_2_	4′-OH	1000	6.00	6.10	5.67	7.46	7.62
11	5-F, 6-OH	4′-OH	3	8.52	8.27	8.32	7.85	7.90
12	5-OH	4′-OH	100	7.00	7.03	6.45	7.15	7.22
13∗	4,7-di(Me),6-OH	4′-OH	100	7.00	**6.96**	**8.03**	6.72	6.74
14	4-OH	4′-OH	190	6.72	6.68	6.16	7.14	6.89
15	7-OH	4′-OH	300	6.52	6.99	7.07	7.63	7.47
16	4,6-di(OH)	4′-OH	350	6.46	6.71	6.72	7.98	8.03
17	5,6-di(OH)	4′-OH	400	6.40	6.33	6.74	6.73	6.75
18	5,7-di(Me),6-OH	4′-OH	500	6.30	6.38	6.46	7.35	7.62
19	4,5-Benzo,6-OH	4′-OH	500	6.30	5.97	5.98	6.78	7.55
20	6-OMe	4′-OMe	300	6.52	6.56	6.82	6.61	6.30
21	5,6,7-tri(OMe)	4′-OMe	350	6.46	6.67	6.25	5.74	5.97
22	6-OMe	3′,4′-OCH_2_O	500	6.30	6.72	7.14	6.29	6.15
23	6-OMe	4′-CH_2_OH	600	6.22	5.87	5.86	6.68	6.40
24	6-OH	H	2.5	8.60	7.92	8.19	7.94	8.24
25∗	6-OH	4′-OH	0.2	9.70	**7.90**	**8.35**	7.90	8.21
26	6-OH	4′-C*≡*CH	0.8	9.10	7.83	8.11	7.81	8.20
27	6-OH	4′-Cl	1	9.00	8.10	8.25	7.41	7.70
28	6-OH	4′-F	2.3	8.64	7.71	8.26	8.08	8.19
29	6-OH	4′-Et	5	8.30	7.81	7.84	7.86	7.94
30	6-OH	4′-CH=CH_2_	7	8.15	7.43	7.97	7.77	7.84
31	6-OH	4′-n-Bu	10	8.00	8.22	7.79	7.84	7.68
32∗	6-OH	4′-i-Pr	30	7.52	**7.81**	**7.76**	7.65	7.62
33	6-OH	4′-Me	50	7.30	7.93	7.98	7.44	7.41
34	6-OH	4′-Ph	100	7.00	7.25	7.72	6.80	7.04
35	6-OH	4′-CH_2_SEt	100	7.00	7.25	7.30	7.49	7.06
36	6-OH	4′-NO_2_	500	6.30	6.65	6.36	6.80	6.55
37∗∗	6-OH	4′-OMe	1000	6.00			7.83	7.97
38∗	6-OH	4′-CONMe_2_	20	7.70	**7.72**	**7.20**	6.67	7.43
39	6-OH	4′-COMe	32	7.49	7.19	7.82	6.95	7.02
40	6-OH	4′-CON(H)Me	40	7.40	6.87	7.29	7.12	7.89
41∗	6-OH	4′-CO_2_Me	50	7.30	**7.23**	**7.33**	7.03	6.79
42	6-OH	4′-CO_2_Et	50	7.30	7.16	7.30	7.77	7.67
43	6-OH	4′-CONH_2_	200	6.70	6.95	7.18	7.42	7.78
44	6-OH	4′-CO_2_H	325	6.49	7.08	6.78	6.92	6.79
45	6-OH	3′-F, 4′-OH	0.3	9.52	9.10	8.78	8.47	8.30
46	6-OH	2′-Me	0.7	9.15	8.85	8.37	8.20	8.25
47	6-OH	3′-Me, 4′-OH	1	9.00	9.31	9.03	8.41	8.39
48	6-OH	2′-Me, 4′-OH	2	8.70	9.29	9.05	8.46	8.40
49	6-OH	2′-OMe, 4′-OH	2	8.70	8.66	8.81	9.65	9.73
50	6-OH	3′-Cl, 4′-OH	2.3	8.64	8.98	9.17	8.55	9.01
51	6-OH	3′-F	2.5	8.60	8.36	8.52	8.51	8.57
52	6-OH	3′-OH	3.2	8.49	8.39	8.39	8.32	8.29
53	6-OH	2′-OH	10	8.00	8.55	8.39	8.41	8.39
54	6-OH	3′,5′-Di(Me), 4′-OH	100	7.00	6.98	7.27	6.58	6.01
55	6-OH	1′-Naphthyl	0.8	9.10	9.39	8.70	8.74	8.17
56	6-OH	4′-OH-1′-Naphthyl	2	8.70	8.74	8.70	8.60	8.35
57	6-OH	*trans*-4′-OH-Cyclohexyl	2	8.70	8.76	8.46	8.28	8.71
58	6-OH	Cyclohexyl	2.5	8.60	8.66	8.47	9.00	9.34
59	6-OH	Isopropyl	3	8.52	8.32	8.48	7.85	7.26
60	6-OH	Cyclopentyl	5	8.30	8.47	8.48	8.10	7.80
61∗	6-OH	4′-Hydroxybenzyl	5	8.30	**8.57**	**8.56**	8.41	8.15
62∗	6-OH	3′-Thienyl	10	8.00	**7.70**	**8.32**	7.47	7.35
63	6-OH	2′-Thienyl	20	7.70	7.64	7.96	7.19	7.24
64∗	6-OH	Ethyl	20	7.70	**7.88**	**8.48**	8.00	8.36
65	6-OH	Methyl	35	7.46	7.30	8.26	7.16	6.68
66	6-OH	2′-Naphthyl	80	7.10	7.09	6.95	8.81	8.70
67	6-OH	4′-Pyridyl	100	7.00	7.80	7.39	7.73	7.96
68	6-OH	4′-Pyridyl N-oxide	100	7.00	7.01	7.01	7.20	7.02

^*^Compounds included in test set of CoMFA and CoMSIA modeling.

∗∗Compounds not included in the training or test set of CoMFA and CoMSIA.

**Table 2:** Statistical data of CoMFA and CoMSIA models on MCF-7 cell inhibition^a^.

	CoMFA	CoMSIA
*q* ^2^	0.519	0.511
ONC	4	4
SEE	0.434	0.443
*r* ^2^	0.816	0.819
*F*	60.320	57.534

Contribution fraction		

*S*	0.515	0.106
*E*	0.485	0.239
HB donor		0.442
HB acceptor		0.212

^a^Abbreviations used are as follows.

*q* ^2^: Leave-one-out cross-validated (LOOCV) correlation coefficient.

ONC: Optimum number of principal components.

*r* ^2^: Non-cross-validated correlation coefficient.

SEE: Standard error of estimate.

*F*: *F*-test value.

*S*: Steric field contribution fraction.

*E*: Electrostatic field contribution fraction.

HB donor: Hydrogen bond donor field contribution fraction.

HB acceptor: Hydrogen bond acceptor field contribution fraction.

**Table 3:** The intercorrelations between the 11 selected features (descriptors) and the activity presented in pIC_50_ for the studied compounds^a^.

	*x*_1_	*x*_2_	*x*_3_	*x*_4_	*x*_5_	*x*_6_	*x*_7_	*x*_8_	*x*_9_	*x*_10_	*x*_11_
pIC_50_	−0.32	0.2	−0.22	−0.25	−0.33	0.11	−0.02	0.19	0.24	−0.22	0.39
*x*_1_	1										
*x*_2_	−0.08	1									
*x*_3_	0.33	−0.01	1								
*x*_4_	0.11	−0.11	−0.1	1							
*x*_5_	0.56	−0.12	0.34	0.43	1						
*x*_6_	0.11	0.18	0.29	−0.01	0.5	1					
*x*_7_	−0.28	0.26	0	−0.2	−0.13	0.43	1				
*x*_8_	−0.12	−0.21	0.05	0.04	0.26	0.63	0.05	1			
*x*_9_	−0.11	−0.03	−0.02	0.37	0.07	−0.02	0.06	−0.01	1		
*x*_10_	0.49	−0.04	0.52	0.29	0.93	0.68	−0.07	0.39	0.06	1	
*x*_11_	−0.43	0.2	−0.12	−0.35	−0.52	−0.25	0.09	−0.07	−0.06	−0.47	1

^a^Descriptors used are

*x*_1_: Complementary information content (CIC) (fast descriptors).

*x*_2_: Estate keys (sums): S_ssCH2 (fast descriptors).

*x*_3_: Estate keys (sums): S_aasC (fast descriptors).

*x*_4_: Estate keys (sums): S_dO (fast descriptors).

*x*_5_: Principal moment of inertia *X* (spatial descriptors).

*x*_6_: Shadow area: *ZX* plane (spatial descriptors).

*x*_7_: Shadow area fraction: *YZ* plane (spatial descriptors).

*x*_8_: Shadow ratio (spatial descriptors).

*x*_9_: Dipole moment *Z* (spatial descriptors).

*x*_10_: SASA (jurs descriptors).

*x*_11_: RPCS (jurs descriptors).

**Table 4:** The model equations generated by support vector regression and linear regression.

		*x*_1_	*x*_2_	*x*_3_	*x*_4_	*x*_5_	*x*_6_	*x*_7_	*x*_8_	*x*_9_	*x*_10_	*x*_11_	Constant	**RMSE**
LR models														

Equation (1)	pIC_50_ =		+0.08*x*_2_	−0.26*x*_3_	−0.24*x*_4_	−0.41*x*_5_	+0.66*x*_6_	−0.48*x*_7_		+0.41*x*_9_		+0.28*x*_11_	+7.54	**0.7364**
Equation (2)	pIC_50_ =		+0.08*x*_2_	−0.26*x*_3_	−0.24*x*_4_	−0.41*x*_5_	+0.66*x*_6_	−0.48*x*_7_		+0.41*x*_9_	−0.01*x*_10_	+0.28*x*_11_	+7.54	**0.7368**
Equation (3)	pIC_50_ =			−0.26*x*_3_	−0.23*x*_4_	−0.42*x*_5_	+0.68*x*_6_	−0.47*x*_7_		+0.41*x*_9_		+0.30*x*_11_	+7.54	**0.7370**
Equation (4)	pIC_50_ =			−0.26*x*_3_	−0.23*x*_4_	−0.42*x*_5_	+0.68*x*_6_	−0.47*x*_7_		+0.41*x*_9_	−0.01*x*_10_	+0.30*x*_11_	+7.54	**0.7374**
Equation (5)	pIC_50 _=			−0.28*x*_3_	−0.22*x*_4_	−0.47*x*_5_	+0.77*x*_6_	−0.51*x*_7_	−0.11*x*_8_	+0.41*x*_9_	+0.03*x*_10_	+0.31*x*_11_	+7.54	**0.7384**
Equation (6)	pIC_50 _=	−0.01*x*_1_	+0.08*x*_2_	−0.25*x*_3_	−0.24*x*_4_	−0.39*x*_5_	+0.67*x*_6_	−0.48*x*_7_		+0.41*x*_9_	−0.03*x*_10_	+0.28*x*_11_	+7.54	**0.7483**
Equation (7)	pIC_50 _=				−0.21*x*_4_		+0.91*x*_6_	−0.56*x*_7_		+0.43*x*_9_	−0.70*x*_10_	+0.30*x*_11_	+7.54	**0.7484**
Equation (8)	pIC_50 _=			−0.26*x*_3_	−0.23*x*_4_	−0.41*x*_5_	+0.69*x*_6_	−0.47*x*_7_		+0.41*x*_9_	−0.03*x*_10_	+0.30*x*_11_	+7.54	**0.7492**
Equation (9)	pIC_50 _=			−0.23*x*_3_		−0.56*x*_5_	+0.84*x*_6_	−0.51*x*_7_	−0.12*x*_8_	+0.34*x*_9_		+0.34*x*_11_	+7.54	**0.7539**
Equation (10)	pIC_50 _=			−0.21*x*_3_		−0.54*x*_5_	+0.73*x*_6_	−0.46*x*_7_		+0.34*x*_9_		+0.33*x*_11_	+7.54	**0.7542**

SVM models														

Equation (1)	pIC_50 _=			−0.28*x*_3_	−0.32*x*_4_	−0.34*x*_5_	+0.62*x*_6_	−0.37*x*_7_		+0.42*x*_9_		+0.32*x*_11_	+7.50	**0.7294**
Equation (2)	pIC_50 _=			−0.30*x*_3_		−0.51*x*_5_	+0.84*x*_6_	−0.48*x*_7_	−0.19*x*_8_	+0.33*x*_9_		+0.38*x*_11_	+7.57	**0.7155**
Equation (3)	pIC_50 _=		+0.09*x*_2_	−0.26*x*_3_	−0.32*x*_4_		+0.74*x*_6_	−0.50*x*_7_	−0.10*x*_8_	+0.54*x*_9_	−0.34*x*_10_	+0.26*x*_11_	+7.55	**0.7104**
Equation (4)	pIC_50 _=			−0.23*x*_3_	−0.35*x*_4_		+0.71*x*_6_	−0.49*x*_7_		+0.57*x*_9_	−0.38*x*_10_	+0.39*x*_11_	+7.60	**0.7408**
Equation (5)	pIC_50 _=	−0.08*x*_1_	+0.06*x*_2_	−0.21*x*_3_	−0.31*x*_4_		+0.67*x*_6_	−0.45*x*_7_	−0.14*x*_8_	+0.43*x*_9_	−0.23*x*_10_	+0.25*x*_11_	+7.56	**0.7430**
Equation (6)	pIC_50 _=		+0.10*x*_2_	−0.28*x*_3_	−0.24*x*_4_	−0.38*x*_5_	+0.61*x*_6_	−0.47*x*_7_	−0.11*x*_8_	+0.46*x*_9_		+0.24*x*_11_	+7.62	**0.7114**
Equation (7)	pIC_50 _=			−0.23*x*_3_	−0.19*x*_4_		+0.59*x*_6_	−0.45*x*_7_	−0.05*x*_8_	+0.47*x*_9_	−0.29*x*_10_	+0.31*x*_11_	+7.57	**0.7298**
Equation (8)	pIC_50 _=		+0.13*x*_2_	−0.20*x*_3_	−0.29*x*_4_		+0.65*x*_6_	−0.45*x*_7_		+0.53*x*_9_	−0.33*x*_10_	+0.33*x*_11_	+7.59	**0.7519**
Equation (9)	pIC_50 _=		+0.03*x*_2_	−0.38*x*_3_	−0.31*x*_4_	−0.29*x*_5_	+0.53*x*_6_	−0.37*x*_7_		+0.53*x*_9_	+0.07*x*_10_	+0.34*x*_11_	+7.62	**0.7523**
Equation (10)	pIC_50 _=						+0.82*x*_6_	−0.53*x*_7_		+0.38*x*_9_	−0.71*x*_10_	+0.37*x*_11_	+7.58	**0.7273**
Equation (11)	pIC_50 _=	−0.05*x*_1_		−0.16*x*_3_		−0.47*x*_5_	+0.69*x*_6_	−0.40*x*_7_		+0.31*x*_9_		+0.33*x*_11_	+7.53	**0.7478**
Equation (12)	pIC_50 _=			−0.28*x*_3_		−0.54*x*_5_	+0.67*x*_6_	−0.40*x*_7_		+0.42*x*_9_	+0.02*x*_10_	+0.34*x*_11_	+7.62	**0.7437**
Equation (13)	pIC_50 _=				−0.25*x*_4_		+0.79*x*_6_	−0.57*x*_7_		+0.48*x*_9_	−0.59*x*_10_	+0.40*x*_11_	+7.57	**0.7462**
Equation (14)	pIC_50 _=			−0.24*x*_3_		−0.48*x*_5_	+0.73*x*_6_	−0.43*x*_7_		+0.40*x*_9_		+0.30*x*_11_	+7.55	**0.7366**
Equation (15)	pIC_50 _=	−0.11*x*_1_	+0.03*x*_2_	−0.25*x*_3_	−0.30*x*_4_	−0.29*x*_5_	+0.50*x*_6_	−0.45*x*_7_		+0.48*x*_9_	+0.03*x*_10_	+0.30*x*_11_	+7.67	**0.7580**
